# Acetazolamide-Induced Periorbital Myokymia: A Case Report

**DOI:** 10.7759/cureus.31920

**Published:** 2022-11-26

**Authors:** Aiswarya Raj, Paul J Alapatt, Paul Johny, Abdurehiman K P, Ashraf V V

**Affiliations:** 1 Department of Neurology, Aster Malabar Institute of Medical Sciences (MIMS), Kozhikode, IND

**Keywords:** : neuro-ophthalmological complications, neurology case report, adverse drug reaction, myokymia, acetazolamide

## Abstract

Acetazolamide, a carbonic anhydrase inhibitor, is primarily used in the treatment of glaucoma, due to its role in decreasing intraocular pressure by lowering the production of aqueous humor. Additionally, by lowering cerebrospinal fluid (CSF) production, it is also used in the treatment of raised intracranial pressure. Drug-induced myokymia has rarely been reported, with known triggers being clozapine, gabapentin and flunarizine, and topiramate. Acetazolamide-induced myokymia itself has only been reported once before, to the best of our knowledge, and the exact mechanism behind this occurrence remains unknown. We, therefore, report a rare case of periorbital myokymia induced by the use of acetazolamide in a patient diagnosed with idiopathic intracranial hypertension. The nature of her symptoms was significant, as they caused her considerable distress, and subsided almost immediately upon discontinuation of the drug.

## Introduction

Myokymia is characterized by spontaneous, involuntary, continuous contraction of a group of muscle fibers in a rhythmic fashion, which is normally visualized as a vermiform movement of the overlying skin [1]. They are brought on by the sequential or simultaneous firing of one, two, or more muscular motor units. According to an electromyography reading, various motor units produce brief, repeating discharges of action potentials that occur in rhythmic or semi-rhythmic bursts. Before the next myokymic discharge happens, there is a brief (0.5-3 seconds) and frequently erratic period of electric silence [2,3]. Facial myokymia occurs either as a persistent rapidly flickering contraction involving the facial musculature (in particular the lower part of the orbicularis oculus) or a contraction that spreads slowly across the face. While varying muscle groups can be affected, eyelid myokymia is the most common cause of facial involuntary muscle movement disorders [4]. The underlying etiology associated with myokymia varies; when associated with muscle cramping and diaphoresis, it may occur as part of Isaacs syndrome, neuromyotonia, episodic ataxias, any condition increasing peripheral nerve excitability (e.g., excessive caffeine intake) and brainstem lesions usually involving the facial nerve are additional diseases linked to myokymia [5].

Acetazolamide is an inhibitor of carbonic anhydrase. Red blood cells and the proximal tubule of nephrons both contain carbonic anhydrase. It works to reabsorb chloride, sodium, and bicarbonate. Inhibiting carbonic anhydrase causes salt, bicarbonate, and chloride, along with excess water to be voided rather than reabsorbed. This leads to decreased blood pressure, intracranial pressure (ICP), and intraocular pressure. Bicarbonate loss also increases the acidity of the blood [6]. Acetazolamide is primarily used in the treatment of glaucoma, due to its role in decreasing intraocular pressure by lowering the production of aqueous humor. Because it blocks choroid plexus enzymes and reduces cerebrospinal fluid (CSF) production, it is also used to treat excessive ICP [7].

Drug-induced myokymia is rare, previously having been reported with clozapine, gabapentin, and flunarizine [8-10]. A suspected case of topiramate-induced eyelid myokymia has also been reported, with the exact mechanism leading to myokymia remaining unclear [11].

Here we present a rare case of periorbital myokymia induced by the use of acetazolamide as treatment in a 24-year-old female diagnosed with idiopathic intracranial hypertension (IIH).

## Case presentation

A 24-year-old patient presented to our outpatient department (OPD) with complaints of episodic bilateral throbbing headache associated with neck pain. She reported two to three episodes per month since the past year, which worsened with movement. When she presented to the hospital, she reported persistent headaches associated with nausea for the past one week. There was no history of vomiting, involuntary movement of her body, limb weakness, blurred vision, or associated aura. She had no significant previous medical history and was not on any regular medication

On clinical examination, the patient had bilateral papilledema. Her neurological examination was normal except for her fundus examination, which showed papilledema. MRI brain was done and showed partial empty sella with prominent peri optic CSF spaces and tortuous optic nerves. A lumbar puncture was done and the opening pressure was 27 cm H2O. The CSF drained was clear and all routine parameters were normal. She was diagnosed as a case of IIH after excluding secondary causes for raised ICP. She was started on acetazolamide 500 mg BD and reported symptomatic improvement.

Within two days of starting acetazolamide, she developed involuntary twitching of her periorbital muscles, suggestive of myokymia. Neurological examination was normal except for the presence of myokymia. The patient consented to be filmed for documentation (Video 1 ).

**Video 1:** Periorbital myokymia, which developed after the patient was started on acetazolamide

Acetazolamide was then temporarily discontinued and the patient immediately reported a complete resolution of symptoms. Acetazolamide was later restarted at a lower dose and she again developed periorbital myokymia. There was, thus, a positive response to the rechallenge of the drug. Acetazolamide was then withheld and her symptoms subsided, which shows a temporal association between acetazolamide and myokymia.

## Discussion

Electromyography (EMG) discharges from a single motor unit can be identified as myokymic discharges if they occur as singlets to multiple waveforms at 5-150 Hz and are repeated at regular intervals. They most probably occur from a local disruption of the nerve's membrane potential. This hypothesis is further supported by the common aetiologies of myokymia, such as Guillain-Barre syndrome and multiple sclerosis [12]. Involvement of potassium conductance in particular has been indicated to play a role in the pathophysiology of this process. In Isaacs-Merton syndrome and Morvan fibrillary chorea, both autoimmune diseases involving antibodies to voltage-gated potassium channel subunits, myokymia is observed [13].

Evidence also demonstrates the presence of potassium channel gene mutations in people with episodic ataxia and myokymia. Mutations in *KCNA1* cause hereditary myokymia with episodic ataxia type 1 [14]. Another rare mutation, *KCNQ2*, causes myokymia with neonatal seizures but without episodic ataxia [15]. It's interesting to note that these individuals' myokymia and episodic ataxia frequency and severity both decreased after receiving acetazolamide medication.

As mentioned, myokymia may occur as part of varying disease pathologies, including multiple sclerosis. The patient's chronic and localized myokymia and lack of involvement of other face muscles suggest that the localization is peripheral [16]. 

Drug-induced myokymia is a rare adverse effect, previously reported with clozapine, gabapentin, and flunarizine [8-10]. A similar presentation was observed in a 47-year-old Iranian woman with a binge eating disorder, who developed eyelid myokymia after being prescribed topiramate for weight management [11]. Two weeks after stopping topiramate, the patient's symptoms started to progressively get better before going away entirely. After she was started on topiramate again, eyelid myokymia reappeared two weeks later.

To the best of our knowledge, there has only been one previously reported case of acetazolamide-induced myokymia [5]. In that report, a 26-year-old woman with IIH developed facial and hand myokymia a few months after being prescribed acetazolamide for IIH. Acetazolamide was then discontinued and the patient reported no further twitching, headache, or impairment of vision.

A similar clinical picture was observed in our patient. After she was diagnosed with IIH, she was started on acetazolamide 500mg BD. However, she developed bilateral periorbital myokymia within two days of the onset of drug administration. There was no definite evidence on MRI suggesting any other cause for her symptoms, and her laboratory parameters including serum electrolytes were all within normal limits. While an EMG was not taken given financial constraints, her clinical picture is suggestive of myokymia. This particular case warrants significant attention, as eyelid myokymia is usually a benign condition; however, in our patient, it caused considerable distress and warranted immediate stoppage of the drug, despite her raised intracranial pressure and then restarted at a lower dose, in view of her current condition.

The exact mechanism for acetazolamide leading to myokymia remains uncertain. Potassium voltage-gated channels are vulnerable to alterations in the membrane potential of the cell. These channels play a crucial part in regulating the nervous system's neuronal excitability [17,18]. Acetazolamide is a carbonic anhydrase inhibitor, which acts by inhibiting reabsorption and thereby promoting the excretion of sodium, bicarbonate, and chloride, leading to diuresis. Carbonic anhydrase inhibitors block fluid production by the choroid plexus (CP), this in turn leads to decreased intracranial pressure, hence explaining its role in the treatment of IIH. Acetazolamide can alter CO2 buildup and membrane hyperpolarization in neurons and glia, and it can also produce metabolic acidosis due to the loss of bicarbonate in urine. It has been theorized that acetazolamide-induced acidosis delays the uptake of potassium by cells, possibly leading to neuronal hyperactivity [5]. This hypothesis is further supported by a possible similar mechanism explaining the previously described case of topiramate-induced myokymia. Topiramate itself has a secondary role as a carbonic anhydrase inhibitor, hence contributing to an environment of metabolic acidosis. Considering the similarities in presentation as well as the temporal relationship demonstrated with both topiramate and acetazolamide in the above-described patients, the most likely mechanism explaining the onset of myokymia as an adverse effect of these drugs is likely to be acidosis-induced nerve hyperexcitability, due to decreased uptake of potassium.

The onset of myokymia and the timing of acetazolamide administration, as well as the amelioration and exacerbation of symptoms after drug cessation and rechallenge, suggest a temporal relationship and, thus, periorbital myokymia must be considered as a rare ocular side effect of acetazolamide.

## Conclusions

Eyelid myokymia, though benign, can cause considerable distress, as was seen in our patient. Keeping in mind the widespread role employed by acetazolamide in neurological treatment, as well as the fact that there are very few existing reports about this condition, it is imperative that clinicians are made aware of this rare adverse effect associated with its use.
